# Atypical Intraepidermal Melanocytic Proliferation Masked by a Tattoo: Implications for Tattoo Artists and Public Health Campaigns

**DOI:** 10.7759/cureus.2975

**Published:** 2018-07-13

**Authors:** Kristina Navrazhina, Barry Goldman, Marie C Leger

**Affiliations:** 1 Medicine, Weill Cornell Medical College, New York, USA; 2 Dermatology, Weill Cornell Medical College/New York Presbyterian Hospital, New York, USA; 3 Dermatology, Weill Cornell Medicine/New York Presbyterian Hospital, New York, USA

**Keywords:** melanoma, tattoo, atypical melanocytic proliferation, early detection, public health

## Abstract

Tattoos have become increasingly popular worldwide. While tattoos carry a minimal risk of complications, previous reports have located malignant melanoma hidden within tattoos. We present a case of an atypical intraepidermal melanocytic proliferation masked by a large tattoo in a 39-year-old Caucasian male. Tattooed skin can be difficult to examine, particularly when the tattoos are dark, pigmented, and extensive. We demonstrate that a careful examination of tattooed skin leads to the early detection of atypical melanocytic proliferations. We present an extensive review of literature related to the relationship between tattoos and skin cancer, as well as public health recommendations for tattoo artists and individuals seeking to obtain tattoos. We urge a vigilant examination of tattooed skin and encourage collaboration between dermatologists and tattoo artists in promoting the detection of suspicious lesions prior and following tattooing.

## Introduction

It is estimated that three in 10 Americans between the ages of 18 and 65 have at least one tattoo [1]. While tattoos may cause inflammatory reactions and introduce infections, they are not considered a risk factor for melanoma development [2-3]. However, tattoos may conceal developing melanomas, with several reports uncovering malignant melanoma hidden within a tattoo [3]. We present a case of a tattoo masking an atypical intraepidermal melanocytic proliferation, a review of reported cases of melanoma in a tattoo, and current research on the relationship between tattoos and melanoma. Importantly, we demonstrate the necessity for skin examinations for patients with tattoos and highlight the implications for education and public health efforts directed at tattoo artists and clients seeking tattoos.

## Case presentation

**Figure 1:**
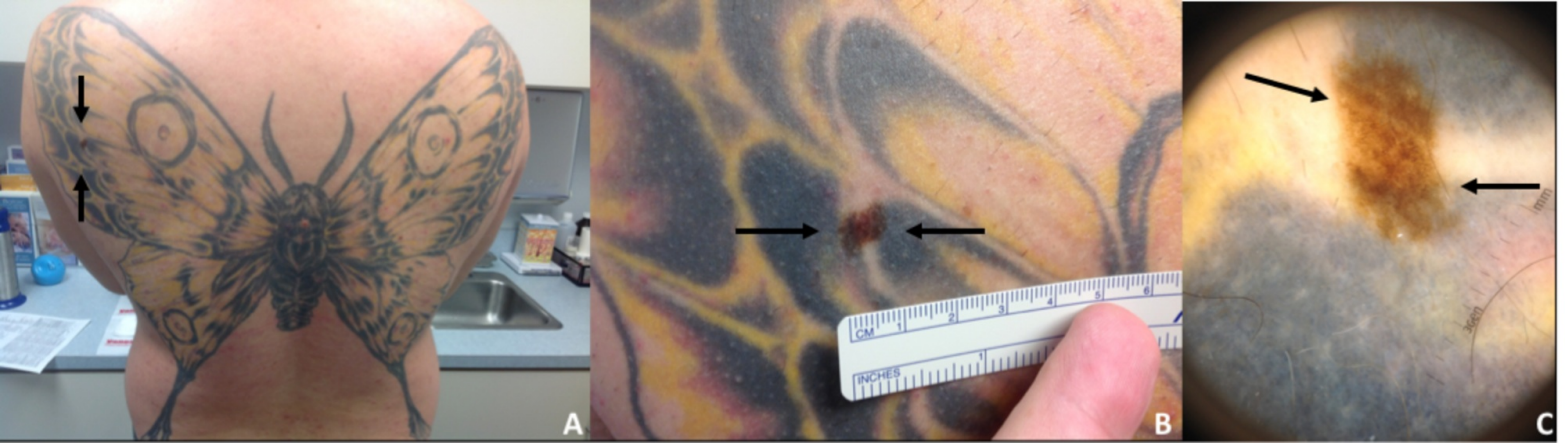
Routine dermatological examination revealed an atypical pigmented lesion within a large tattoo (A) Butterfly tattoo on the back with a pigmented lesion within the tattoo (black arrows). (B) Up-close photograph of the 8-mm, flat, brown papule on the back (black arrows). (C) Dermoscopy of the asymmetrical, pigmented lesion demonstrated irregular streaks and multiple colors (black arrows).

A 39-year-old Caucasian male presented for a dermatological evaluation. His exam demonstrated an 8-millimeter flat, brown papule within a large tattoo covering most of the upper back (Figure 1). The patient was uncertain regarding the lesion’s initial presentation, the pattern of its growth and appearance, how long the lesion has been present, and whether the lesion was present at the time of tattooing. A shave removal of the lesion was performed.

**Figure 2:**
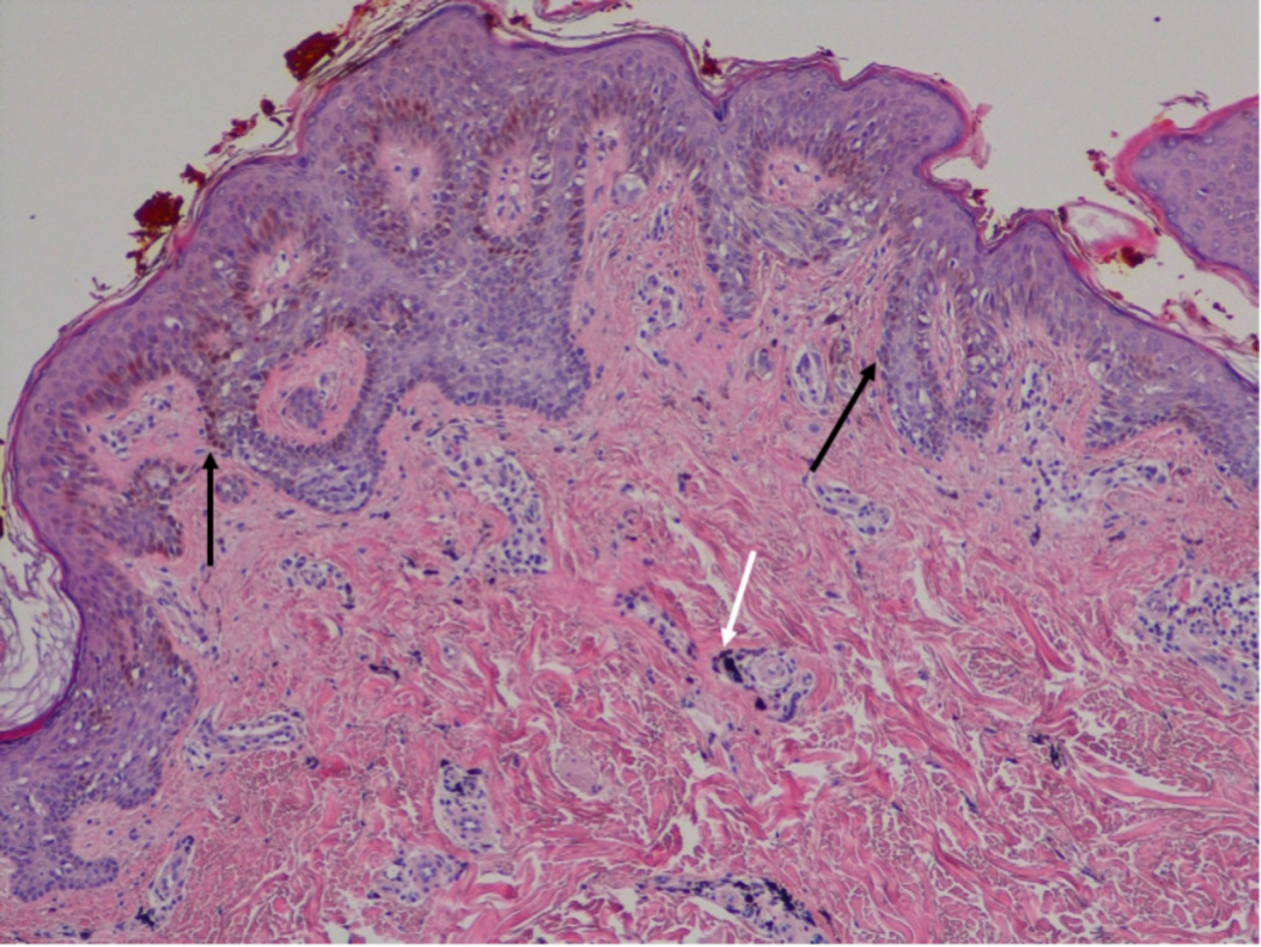
Microscopic examination demonstrated an atypical intraepidermal melanocytic proliferation There is a high-density lentiginous and nested intraepidermal melanocytic proliferation (black arrows). The melanocytes are large with conspicuous nucleoli. A few, small dermal nests composed of banal melanocytes are identified. Scattered clusters of opaque black pigment are present within the superficial dermis (white arrows).

**Figure 3:**
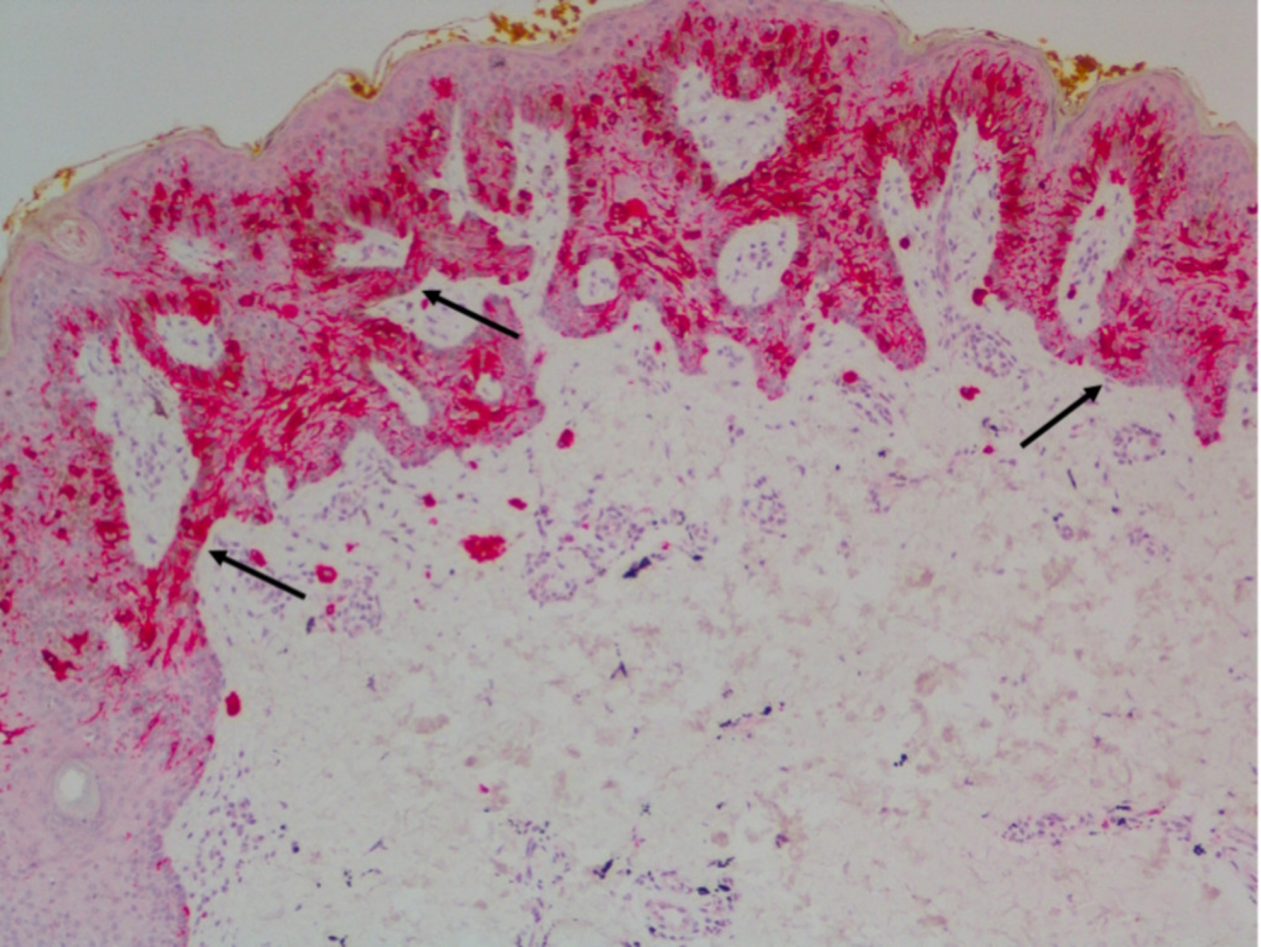
Melan-A immunohistochemical stain confirmed intraepidermal melanocytic proliferation Melan-A immunohistochemical stain revealed a high-density intraepidermal melanocytic proliferation composed of confluent nests and scattered pagetoid solitary melanocytes (black arrows).

**Figure 4:**
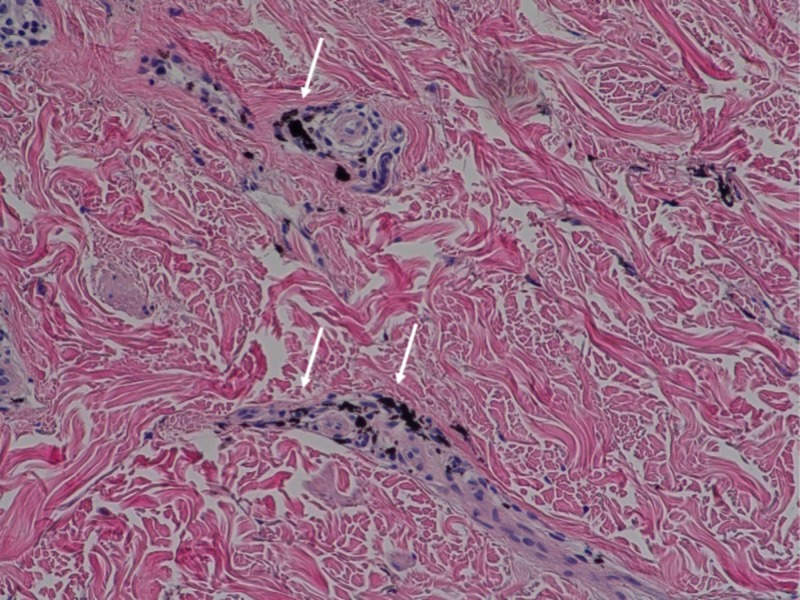
Microscopic examination identified black tattoo pigment Opaque black tattoo pigment is present in the dermis (white arrows).

The original excision showed an atypical intraepidermal melanocytic proliferation (Figures 2-3). The dermis contained scattered foci of tattoo pigment and fibrosis (Figures 3-4). An early evolving melanoma in situ could not be excluded. The lesion was re-excised with a 5-millimeter margin. No residual melanocytic lesion was detected on examination; only scar tissue was present. The patient has healed well since then. He continues to have his skin monitored.

## Discussion

Tattoos carry minimal risk, with previous studies reporting short-term (lasting <4 months) reaction rates of 4.3% following tattooing [4]. The most common short-term complications of tattoos include swelling, itching, and scabbing [4]. Other complications of tattoos include acute and chronic infections, ranging from local responses to systemic chronic infections, and allergic reactions, such as contact dermatitis from ink pigments, as well as urticarial and granuloma formation [5-6].

The presence of tattoos can hide developing malignancies. Malignancies such as squamous cell carcinomas, basal cell carcinomas, and keratoacanthomas have been more frequently diagnosed within a tattoo [3]; however, there have been only 17 reports in the English literature of melanoma arising in tattoos [7]. A review of case reports demonstrates that melanoma and basal cell carcinoma tend to occur on dark-colored tattoos (black or dark blue pigment), whereas squamous cell carcinoma, keratoacanthomas, and benign pseudoepitheliomatous hyperplasia have been linked to red tattoos [8]. In addition to masking suspicious nevi and thus making it difficult for dermatologists to assess new or changing nevi, the ink pigment has been reported to mimic a metastatic disease in the sentinel lymph node biopsy of patients diagnosed with melanoma [7-8]. While tattoos are not considered a risk factor for melanoma development, several multifactorial hypotheses have emerged regarding a potential link between tattooing and skin cancer [2-3]. The trauma of puncturing the skin has been linked to keratoacanthoma development in the first year following tattooing; however, recent evidence suggests that trauma does not lead to melanoma development in tattoos [2,8]. It has been suggested, but not established, that the process of tattooing can cause the seeding of existing melanoma cells. Therefore, patients should be encouraged to undergo a skin examination by a dermatologist prior to receiving a tattoo.

There is a lack of definitive data outlining the role of tattoo ink pigment in skin cancer pathogenesis.In viv*o* studies in immunocompetent hairless mice, an accepted model for studying human tattooing, have demonstrated that while red tattoos alone do not induce cancer, red ink may act as a co-carcinogen in combination with ultraviolet radiation (UVR) to promote quicker onset and tumor growth [9]. Interestingly, black tattoos have been shown to delay squamous cell carcinomas (SCCs) following ultraviolet radiation (UVR) exposure [10]. The protective effect of black inks may be due to black ink absorbing more UVR in the dermis, thereby decreasing the amount of backscattered radiation reaching the epidermis [9]. Nevertheless, an independent analysis of 19 commercially available black tattoo inks identified varying concentrations of hazardous polycyclic aromatic hydrocarbons, some of which have been reported to be carcinogenic [11].

While there is no definitive in vivo data implicating tattoo pigments in skin cancer development, there is a concern regarding the carcinogenic potential of tattoo inks and their photo degradation-induced byproducts. The content of tattoo inks is not standardized worldwide and, frequently, tattoo ink manufacturers do not provide a detailed list of components in the ink [10]. In the past 15 years, organic ingredients, such as azo dyes, have started to replace the potentially carcinogenic metallic salts [3,8]. However, studies have shown that when exposed to sunlight or lasers, azo pigments degrade into various compounds, some of which, such as 2-amino-4-nitrotoluene, 3,3’-dichlorobenzidine, and o-toluidine, have a toxic and carcinogenic potential [3,12]. A previous review of the literature showed that a majority of melanomas arising within tattoos occurred on sun-exposed areas [2]. Although this suggests there may be a link between sun exposure and melanoma development in tattoos, it remains unclear whether tattoos accelerate melanoma development in the setting of ionizing radiation and ultraviolet light.

The incidence of melanoma and melanocytic proliferation is increasing, necessitating increased vigilance. Tattoo artists play a pivotal role in counseling patients on the aftercare and adverse effects of tattooing. A previous report showed that only 55.2% of surveyed tattoo artists either look out or point out atypical moles while tattooing [6]. Our case highlights that a close examination of tattoos can detect the development of melanoma in early stages. When educated by dermatologists, tattoo artists can have a role in identifying suspicious lesions prior to tattooing. Tattoo artists should learn about individuals’ family history of skin cancer prior to tattooing. Nearly 90% of surveyed tattoo artists have expressed an interest in further education about skin conditions, and our case highlights the need for a prospective study to identify the role of dermatological education in the practice of tattooing [6].

Individuals with numerous nevi, atypical mole syndrome, or a family history of melanoma should have a dermatological exam prior to tattooing and consistent skin monitoring following tattoo placement. As part of preventative care, dermatologists should inquire if patients with the aforementioned risks are considering getting a tattoo, as patients may be unaware of the dermatological implications of tattooing. All individuals with tattoos should undergo a close monitoring of tattoos and regular skin examinations to facilitate the early diagnosis and treatment of developing skin malignancies.

## Conclusions

Tattoos may mask the development of skin cancer, particularly in large and darkly pigmented graphics. Here, we present a case of an atypical melanocytic proliferation in a large tattoo. Our report demonstrates that the careful examination of tattoos can lead to the successful detection of early-stage skin cancer development in patients with extensive tattoos. With the increased popularity of tattoos, we urge both patients and health-care providers to be vigilant regarding the examination of tattooed skin.
